# Liquid Biopsy in Melanoma: Significance in Diagnostics, Prediction and Treatment Monitoring

**DOI:** 10.3390/ijms22189714

**Published:** 2021-09-08

**Authors:** Paula Kamińska, Karolina Buszka, Maciej Zabel, Michał Nowicki, Catherine Alix-Panabières, Joanna Budna-Tukan

**Affiliations:** 1 Department of Histology and Embryology, Poznan University of Medical Sciences, 60-781 Poznan, Poland; kaminska.p19@gmail.com (P.K.); karolina.anna.buszka@gmail.com (K.B.); mnowicki@ump.edu.pl (M.N.); 2 Department of Anatomy and Histology, Collegium Medicum, University of Zielona Góra, 65-046 Zielona Góra, Poland; mazab@ump.edu.pl; 3 Laboratory of Rare Human Circulating Cells (LCCRH), University Medical Centre of Montpellier, 34093 Montpellier, France; c-panabieres@chu-montpellier.fr; 4 CREEC/CANECEV, MIVEGEC (CREES), University of Montpellier, CNRS, IRD, 34000 Montpellier, France

**Keywords:** circulating tumor cells (CTCs), liquid biopsy, malignant melanoma, circulating melanoma cells (CMCs), metastasis, targeted treatment, immunotherapy

## Abstract

Liquid biopsy is a common term referring to circulating tumor cells and other biomarkers, such as circulating tumor DNA (ctDNA) or extracellular vesicles. Liquid biopsy presents a range of clinical advantages, such as the low invasiveness of the blood sample collection and continuous control of the tumor progression. In addition, this approach enables the mechanisms of drug resistance to be determined in various methods of cancer treatment, including immunotherapy. However, in the case of melanoma, the application of liquid biopsy in patient stratification and therapy needs further investigation. This review attempts to collect all of the relevant and recent information about circulating melanoma cells (CMCs) related to the context of malignant melanoma and immunotherapy. Furthermore, the biology of liquid biopsy analytes, including CMCs, ctDNA, mRNA and exosomes, as well as techniques for their detection and isolation, are also described. The available data support the notion that thoughtful selection of biomarkers and technologies for their detection can contribute to the development of precision medicine by increasing the efficacy of cancer diagnostics and treatment.

## 1. Introduction

The increasing incidence of cancer remains a challenge of modern medicine [1]. Among the commonly diagnosed malignancies, melanoma, derived from epidermal improperly proliferating melanocytes, is characterized by relatively high mortality, which may be dependent on such risk factors as exposure to UV rays, high amounts of melanocytic nevi, melanoma cases in a patient’s family, and specific phenotype (fair skin, eyes, sunburns easily, etc.) [2].

**Figure 1:**
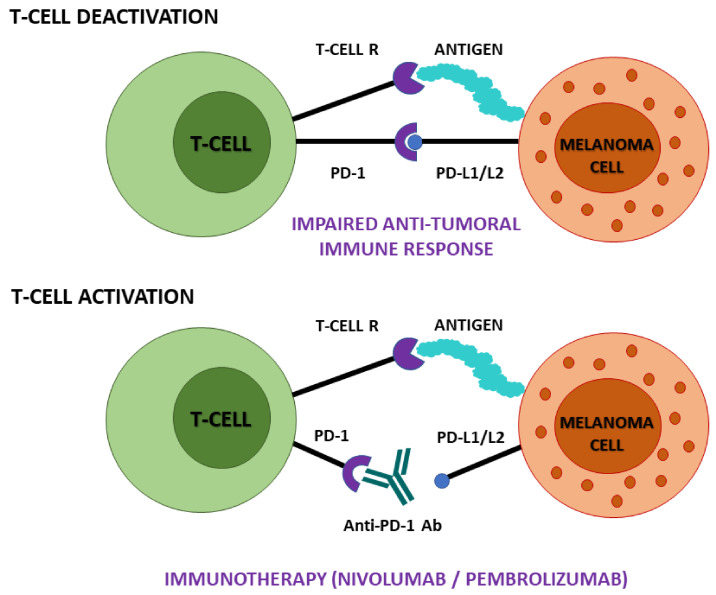
Immunotherapy of PD-1–PD-L1/L2 axis blockade—mechanism of action. Anti-PD-1 monoclonal antibodies nivolumab and pembrolizumab enhance the anti-cancer response by blocking the binding of PD-1 to PD-L1/L2, thus activating T-cells to eradicate cancer cells. Abbreviations: PD-1, programmed cell death 1; PD-L1/L2, programmed cell death ligand 1/2; T-cell R, T-cell receptor; Ab, antibody.

Conventional melanoma treatments start from surgical lesion removal and biopsy assessment [3]. As the currently used methods of cancer treatment, including chemotherapy and radiotherapy (RT), are often less effective, invasive, and cause significant complications for patient health and wellbeing, significant effort is placed on the development of more targeted approaches [4]. There are several immune therapies that have successfully been used in the treatment of malignant melanomas, solely as well as combined with other approaches, like cytotoxic T lymphocyte-associated antigen 4 (CTLA-4) inhibitor ipilimumab [5] or programmed cell death protein 1 (PD-1) inhibitors nivolumab and pembrolizumab (Figure 1) [6,7].

**Figure 2:**
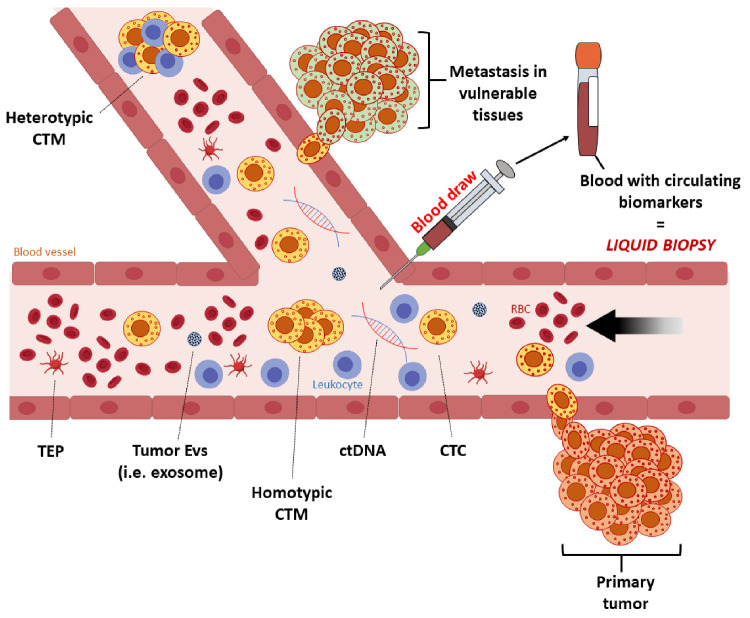
A schematic of liquid biopsy in a patient with a solid tumor. Tumor cells circulate in blood, with those that are more aggressive (metastasis-initiator cells) settling in targeted distant organs and initiating metastasis. Blood collected from cancer patients contains circulating tumor cells (CTCs), clusters or circulating tumor microemboli (CTM), proteins, circulating cell-free tumor DNA (ctDNA), extracellular vesicles (EVs), such as exosomes, and tumor educated platelets (TEPs). These complementary circulating biomarkers can be detected and provide real-time information on tumor progression, prognosis, and treatment response. Abbreviations: CTCs, circulating tumor cells; ctDNA, circulating tumor DNA; CTM, circulating tumor microemboli.

The recent statistics for this disease show a promising trend—mortality is steadily declining, presumably as a result of the approval of new therapeutic methods, especially immunotherapy. Nevertheless, the appearance of new cases is still relatively high [8]. A standard diagnostic procedure involves a panel of tumor markers and a biopsy of the lesion. However, due to their invasiveness, traditional tumor biopsies are not suitable for continuous disease monitoring [9]. Research aimed at overcoming this obstacle led to the introduction of so-called liquid biopsies, minimally invasive and cost-effective diagnostic methods with a high level of sensitivity [10,11,12,13]. The term, first used by Pantel and Alix-Panabières [14,15], refers to a blood test that provides comparable or even more specific information than the classic tissue biopsy. These could include the concentration of proteins, or the presence and expression of tumor markers, with the latter mainly consisting of circulating tumor cells (CTCs) and their products [16]. Many current reports indicate CTCs, as well as circulating tumor DNA (ctDNA), circulating cell-free mRNA, and exosomes as promising biomarkers for diagnosis, control of disease progression, or evaluation of treatment effectiveness in many types of cancers (Figure 2) [11,17,18].

Differences in disease outcome, prognosis, and the availability of effective targeted therapies require tools for better stratification, selection of the optimal time to start the therapy, and drug resistance monitoring. The diversity of melanoma in terms of surface marker expression makes reliable detection a challenge and thus inspires the use of different detection methods [19].

## 2. Liquid Biopsy in Melanoma

### 2.1. Circulating Melanoma Cells (CMCs)

CTCs in melanoma, known as circulating melanoma cells (CMCs), were described in the early 1990s [20]. Like all circulating tumor cells, CMCs descend from the primary tumor [21]. For an invasion to occur, cancer cells must change their nature periodically to suit the current environment. Therefore, CTCs undergo reversible phenotypic changes enabling epithelial-mesenchymal transition (EMT) [22]. During this process, changes in the cytoskeleton occur, together with a loss of adhesive proteins, which increases the mobility of cancer cells [23]. This enables their transport from the primary location to other, distant and susceptible tissues, as well as the formation of micro- and/or macro-metastases. These processes suggest a large heterogeneity of CMCs [24], making it necessary to constantly search for new and improved methods of their use in melanoma diagnostics. Another disadvantage is the relatively low number of CMCs in peripheral blood, with 1–3 CMCs corresponding to 5 billion blood cells [25]. Thus, an enrichment stage increasing the concentration is highly recommended for an optimized CMC detection [18].

In contrast to other cancer types, which frequently establish CTMs (circulating tumor microemboli) in metastatic stages, CMCs occur mostly individually in the bloodstream of patients with melanoma [26]. However, Long et al. confirmed the existence of CTMs in metastatic melanoma patients. The presence of such clusters reflects the increased metastatic potential and higher invasiveness of cells, affecting the overall survival. This occurrence could result from the heterogeneous phenotype of cells enclosed in CTMs [27], as well their higher resistance to apoptosis [28].

CMCs express a variety of surface antigens used in various isolation and detection techniques, including melanoma cell markers, e.g., MCAM (melanoma cell adhesion molecule, MCAM/MUC18/CD146) [25], MART-1 (melanoma antigen recognized by T cells 1/Melan-A) [29], MAGE-A3 (melanoma antigen A3), PAX3 (paired box 3), HMW-MAA (human high-molecular weight melanoma-associated antigen) [30] and GM2/GD2 (gangliosides) [31]. Importantly, none of the mentioned antigens’ expression is restricted to melanoma cells. Some of them can be found on benign melanocytes and nevi (MCAM, HMW-MAA, MART-1), spermatocytes and other cancer cells (MAGE-3), and endothelial cells (MCAM), while others on normal tissues (PAX3, HMW-MAA) [32]. Thus, antigen-based detection technologies carry the risk of non-specific staining, and results need to be analyzed in relation to clinical data.

Another important melanoma-specific marker is the S100 protein. The S100 protein family consists of 21 structurally similar but functionally different proteins. Their abnormal expression is characteristic for melanoma, with different types of this disease characterized by a different set of expressed proteins, stimulating processes associated with cancer progression [33]. The presence of those proteins confirms the viability of cancer cells that participate in the malignant invasion, making S100 proteins useful in disease monitoring and prognosis [20].

### 2.2. Circulating Tumor DNA (ctDNA)

Apart from CMCs, the “liquid biopsy” also includes other blood-based biomarkers. Among them, circulating cell-free DNA (cfDNA), first described by Mandel and Metais in 1948 [34], was found to be a good source of information about specific mutations and gene alterations. This double-stranded DNA fragment forms a protein complex, with its length ranging from 18 to 10,000 bp [35]. Its presence results from physiological cell functions, such as secretion, apoptosis, or necrosis [36], and can be found in different types of body fluids, including plasma [34]. Nevertheless, it was discovered that cancer patients exhibit higher cfDNA blood levels than healthy controls [18,37]. Hence, cancer-derived cfDNA, referred to as circulating tumor DNA (ctDNA), has attracted significant research attention. Comparing to cfDNA, ctDNA is shorter, around 134–144 bp [38], and usually fragmented because of the presence of nucleosomal single, double, or triple complexes. As blood collection is minimally invasive, ctDNA samples could be collected to examine changes in their quantity and composition overtime, being a useful tool for cancer detection and monitoring [37]. Although ctDNA is quickly degraded by nucleases, with a half-life of less than 2 h [39], its amount was shown to correlate with the stage of the disease, size of the tumor, and the presence of metastases, also in melanoma [40,41].

Since cutaneous melanoma is regarded as a tumor of high tumor mutation burden (TMB) [42], detection of tumor-specific changes, such as variations in DNA integrity, chromosomal rearrangements, or mutations in oncogenes and tumor suppressor genes [35], allows for early detection of the primary disease, adjustment of targeted therapy, treatment progress monitoring, estimation of recurrence risk, and detection of treatment–resistance mutations [43,44,45,46].

Genes subjected to somatic mutations testing include *BRAF*, *CDK4*, *GNAQ*, *JAK2*, *KRAS*, *MAP2K1*, *NF1*, *NRAS*, and *STAT1* [40], with most commonly detected mutations affecting *BRAF* (V600E/K/R, L597R/S), *NRAS* (Q61K/L/R, G12D), and *KIT* (L576P). Among them, the most common is the BRAF oncogenic driver V600 mutation, as it is found in 40–50% of cutaneous melanomas [47].

Since hypermethylation at the promoter region of tumor suppressor genes, e.g., *RARB2*, *TFPI2*, *RASSF1A*, *MGMT*, and *PTEN*, helped to distinguish melanoma patients from healthy individuals [48], epigenetic changes in cfDNA may also serve as diagnostic markers in melanoma [49].

Thus, ctDNA is a promising biomarker for the detection of crucial mutations and epigenetic changes, helping to manage patients with melanoma in diagnostic and therapeutic aspects. Defining the profile of genetic alterations in reference to neoplasm progression is of the greatest importance in terms of patient stratification improvement [50].

### 2.3. Melanoma-Derived Exosomes

Melanoma can modulate its microenvironment through various kinds of factors, promoting its growth and formation of metastasis. Among direct cellular interactions, secretion of signaling factors (cytokines, chemokines, growth factors) and shedding of extracellular vesicles (EVs), are among the most promising sources of cancer information [51]. EVs are commonly divided into shedding vesicles (SVs) and exosomes, depending on the subcellular mechanism of formation and release [52]. Exosomes are nano-sized vesicles of about 100 nm, surrounded by a lipid bilayer [51,53], released into the external microenvironment through the fusion of subcellular multivesicular bodies with the plasma membrane [54]. Although they are found in large quantities in most body fluids, e.g., blood, urine, and breast milk [55,56], exosomes are also involved in vascular leakage in pre-metastatic sites, which plays an important role in the formation of the pre-metastatic niche [57].

The ability of exosomes to participate in intercellular communication, as they partake in the transfer of proteins, RNA, or lipids, indicates their potential involvement in mechanisms of cancer origin [53,58,59]. The evidence that melanoma exosomes can actively communicate with nearby melanocytes comes from the study of Xiao et al., in which exosomes derived from an A375 melanoma cell line and primary normal human epidermal melanocytes (NHEM cell line) were marked with a red and green dye, respectively. During the time of observation, the red-labeled exosomes merged with the membranes of primary green-labeled melanocytes, with a change of color to orange observed [60].

Their molecular cargo, consisting of unique mRNA, miRNA, and proteins, was found both in melanoma cell lines [61] and blood of advanced-stage melanoma patients [62].

A connection has been reported between exosomes and drug resistance in many types of cancers, including melanoma [63,64]. PDGFRβ, belonging to the group of tyrosine kinase receptors, is overexpressed in melanoma and was identified as a drug resistance factor. Exosomes were shown to transfer PDGFRβ to melanoma cells, activating the phosphatidylinositol-3-kinase (PI3K-AKT) signaling pathway, involved in the growth and survival of cancer cells, consequently lowering the sensitivity to BRAF inhibitors [65].

Since exosomes carry a molecular “fingerprint” of the cell of origin, they could deliver invaluable information about the cancer status, making them prospective biomarkers for melanoma diagnosis or prognosis [66].

## 3. Technologies for Liquid Biopsy in Melanoma

### 3.1. CMC Enrichment and Detection

Most commonly, the first step of CMC detection considers their enrichment, significantly increasing cell concentration in the sample and removing unnecessary cells through, e.g., sample centrifugation and positive or negative selection [18,67]. Then, isolation and characterization can be performed based on the physical or biological features of the cells [18]. Physical methods depend on size, density, deformability, and electric charges, whereas biological methods are based on protein marker expression [68].

Physical methods might be useful regardless of population heterogeneity [68], as was proven by Aya-Bonilla et al., who found various CMC subpopulations in metastatic melanoma patients [69]. The more advanced alternative is based on isolation by size of epithelial tumor cells (ISET), being also suitable for cells of non-epithelial origin like CMCs. This single step size-based enrichment ensures the integrity of isolated cells, enabling their further testing using qRT-PCR or immuno-cytochemistry. The essential advantage of ISET is its detection sensitivity of 1 CMC per 1 mL of blood [26]. Another marker-independent enrichment option is the OncoQuick^®^ (Greiner Bio-One, Kremsmünster, Austria) system. This methodology uses the concept of gradient centrifugation with the use of a porous membrane device. The initial cell fraction is enriched 400–500 times, which translates to high CMC yields, and assessed by increased RNA levels compared to the control group.

In turn, only targeting melanoma-specific markers leads to selective isolation and reliable analysis of CMCs [70]. For this purpose, the positive immunomagnetic approach is commonly used. The principle is based on the application of antibodies labeled with magnetic beads against antigens of interest, enabling the extraction of CMCs in the magnetic field. Among targeted proteins, HMW-MAA [71], MCAM, ABCB5 (ATP-Binding cassette subfamily B member 5), CD271 [70], CD133, and nestin [72] are often selected, often in combination to deal with the problem of heterogeneity. Several technologies based on the abovementioned principle have been developed so far, e.g., EasySep^®^ (Stemcell Technologies, Vancouver, BC, Canada) [72], MACS^®^ separator (Miltenyi Biotec, Bergisch Gladbach, Germany) [73], Dynabeads^®^ Antibody Coupling Kit (Invitrogen, Waltham, MA, USA) [70], and Dynabeads^®^ CELLection Pan Mouse IgG Kit (Invitrogen) [74]. Although very selective, it has to be noted that positive isolation can omit cells not expressing certain antigens, thus some CMC subpopulations.

On the contrary, immunomagnetic negative selection depletes all leukocytes through CD45 targeting or endothelial cells through CD34 targeting [75,76], leaving remaining CMCs intact and suitable for phenotyping and molecular analysis. However, the risk of losing CMCs with the CD45 population exists [74], showing that neither approach is perfect. Nevertheless, the ability to isolate even relatively small subsets of CMCs was shown to be clinically relevant [70].

**Figure 3:**
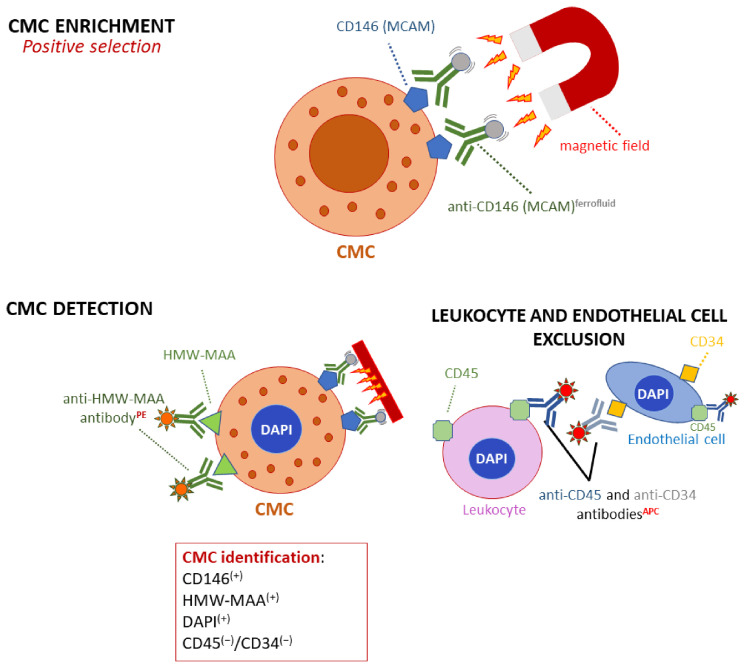
CellSearch^®^ strategy to enrich and detect CMCs in patients with a melanoma. The positive CMC enrichment is based on the expression of CD146 by melanoma cells. Subsequently, CMC detection is based on the expression of the high molecular weight melanoma-associated antigen, as well as the presence of a nucleus (visualized using DAPI). The exclusion markers are (i) CD45, specific for leukocytes and some endothelial cells and (ii) CD34, expressed by endothelial cells. As endothelial cells also express CD146, CD34 is important to consider to discriminate these cells from CMCs that are CD34^(−)^. Abbreviation: MCAM, melanoma cell adhesion molecule; HMW-MAA, high molecular weight; PE, phycoerythrin; APC, allophycocyanin.

As far as detection is concerned, widely used strategies based on the use of EpCAM (epithelial cell adhesion molecule) and other epithelial markers are not useful in this context, as their expression does not occur in melanoma cells [25]. Instead, CMC isolation methods based on an immunomagnetic approach utilize specific melanoma markers. CellSearch^®^ is an example of such methods, designed for isolation of CMCs based on an immunomagnetic capture of cells expressing MCAM (CD146) on their surface from whole blood samples (Figure 3) [24].

Another group of methods contains modifications of flow cytometry (FCM), such as immunomagnetic cell sorting [73], fluorescent-activated cell sorting (FACS) [72,77], or photoacoustic FCM. Goldschmidt and Viator employed the latter approach for CMC capture, detection, and isolation. The method allowed for the detection of individual melanoma cells, which were highly viable and therefore could be successfully subjected to further analyses [78].

The viability of CMCs is a significant biological feature, corresponding to the extent of metastatic potential used in prognosis estimation [18]. Epithelial ImmunoSPOT (EPISPOT) assay allows for the detection of viable cells (also of non-epithelial origin) due to their ability to produce proteins, in case of CMCs, particularly S100. Antibodies are oriented against protein products, each being a “fingerprint” of a living cell. This is a major advantage over CellSearch^®^, which detects both viable and dead cells, potentially making EPISPOT a better method for metastasis progression prediction [79].

Lastly, RT-PCR methods are frequently used for indirect detection of CMCs, through amplification of mRNA (e.g., tyrosinase, Melan-A, MART-1, PAX) extracted from blood [68]. Tyrosinase expression is typical for melanoma cells and melanocytes, with neither of them found in healthy patients’ blood [80,81]. Research by Smith et al. indicated high sensitivity of this method for the detection of cancer cells [82], which was confirmed in later studies [80]. Importantly, it was found that combining various markers in a so-called multimarker RT-PCR increases the sensitivity of this approach and helps to mitigate the effects of CMC heterogeneity, giving RT-PCR methods an advantage over selective immunomagnetic cell capturing.

In turn, DNA sequencing gives the possibility to detect genetic changes characteristic for melanoma. Certain abnormalities occur solely in cancer cells and might therefore be considered as tumor markers, with their tracking enabled via PCR or next-generation sequencing (NGS) methods [83,84]. Thus, melanoma type-specific mutations are crucial for detection technique accuracy. The most common mutations in the CSD type are associated with *NF1* (neurofibromin 1), *NRAS*, *BRAF*, or *KIT* (KIT Proto-Oncogene, Receptor Tyrosine Kinase) genes [85]. In turn, *BRAF* mutations are the dominating genetic causative factor in superficial spreading melanoma (SSM) [85], whereas for ALM, typical mutations can be found in the *BRAF*, *NRAS*, *KIT*, and *NF1* genes [86].

### 3.2. ctDNA Detection

The amount of ctDNA present in the circulation of melanoma patients at the onset of the disease is small and accompanied by DNA from non-cancerous cells, thus proper identification of disease in its early stage is often hampered [87]. To isolate and analyze ctDNA from plasma samples, sensitive molecular techniques are used, often preceded by detection of primary tumor-specific mutations [88,89].

Following the tumor characterization and pre-identification of gene targets, isolated ctDNA can be subjected to PCR-based methods, which can be divided into targeted and nontargeted sequencing. The former is highly sensitive and predominantly include droplet digital polymerase chain reaction (ddPCR) and bead–emulsion–amplification–magnetics (BEAMing) technology, both based on quantification following DNA amplification in water-oil droplets, with comparable and sufficient reproducibility [90]. Most studies applied ddPCR, as it enables detection of *BRAF* and *NRAS* gene mutations, allowing patient response to immunotherapy to be evaluated, as well as recurrence and pseudo-progression to be detected [91]. Studies of Wong et al. [92], Váraljai et al. [93], McEvoy et al. [94], and Forthun et al. [83] confirmed ddPCR validity through quantification of *TERT* promoter and *BRAF*/*NRAS* mutations. The mutational profile, together with ctDNA levels, was associated with disease progression and is useful for monitoring bevacizumab treatment response. The safe-sequencing system (Safe-SeqS), cancer personalized profiling via deep sequencing (CAPP-Seq), and tagged-amplicon deep sequencing (TAmSeq) are other examples of targeted techniques [95].

In turn, next-generation sequencing (NGS) is carried out via whole-genome sequencing (WGS) whole-exome sequencing (WES), whole transcriptome shotgun sequencing (WTSS), targeted (TS) or candidate gene sequencing (CGS). While these methods enable a complex analysis of genetic changes in ctDNA, their lower sensitivity requires a higher concentration of ctDNA, which usually makes them unsuitable for patients without metastases [96,97].

Therefore, currently developed approaches are focused on the successful detection of mutations and epigenetic changes in ctDNA of low concentration. In melanoma, widely distributed mutations in *BRAF* and *NRAS* genes make most of the technologies suitable for analysis. However, in the case of patients harboring *BRAF* and *NRAS* wild-types, analysis is far more complicated. The solution for this problem may be newly introduced approaches based on MALDI-TOF mass spectrometry [89] or a combination of surface-enhanced Raman spectroscopy with PCR [98]. However, their utility is yet to be confirmed.

Gorges et al. presented the advantages of combined analysis of CMCs and cfDNA, which provides a significant amount of information about tumor profile from a singular blood sample [99]. Similarly, in the research of Salvianti et al., CMCs, as well as cfDNA, were used to detect mutated *BRAF*(*V600E*) via qRT-PCR and were validated using ICE COLD PCR with Sanger-sequencing. The authors propose the combined analysis since cfDNA is not tumor-specific, and CMCs might provide useful information about tumor heterogeneity [100].

### 3.3. Exosome Isolation

Although the distinction between tumoral and physiological exosomes is hardly impossible, it is believed that cancer patients present higher content of exosomes, which could serve as a reliable biomarker for tumor management [101]. Exosome analysis differs from the abovementioned techniques, as obtaining exosomes carrying particular content depends on the isolation method used [102].

These generally involve a series of ultracentrifugation steps of different speeds, lasting several hours, to exclude cells, their residues, and, most importantly, to separate exosomes from other types of EVs. This method provides high recovery, along with improved specificity in comparison to single speed non-differential ultracentrifugation [103]. Tubes in more advanced ultracentrifugation are additionally equipped with a density gradient enabling separation of even slightly different EVs based on their density [104]. Combining both methodologies allows cost-effective and efficient purification of a satisfactory number of exosomes to be used for further studies, focusing on their cargo, e.g., proteins and miRNAs [54]. To improve enrichment via ultracentrifugation, it can be preceded by a filtration step, using a 0.22 μm filter, removing cell debris, protein aggregates, and lipoproteins [105].

The size of exosomes is the feature used for their enrichment via ultrafiltration or size exclusion chromatography [106,107]. The procedure relies on the application of Sepharose-filled columns of certain porosity, allowing separation of exosomes from other EVs. Although they are characterized by intermediate recovery, possibly resulting in contamination with other EVs types and proteins [104], the use of both methods for more effective isolation can be beneficial. As an example, Shu et al. combined ultrafiltration and size exclusion chromatography, which allowed more exosomes to be isolated than in any of the individual approaches [102].

Purification effectiveness is usually evaluated by the presence of markers specific for endosomal plasma membrane and cytoplasmic components enclosed within vesicles, as well as a simultaneous absence of contaminants like albumin and A1/2 and B apolipoproteins [104].

Yet another isolation method is based on immunoaffinity, directly retrieving exosomes expressing CD9, CD63, or CD81 on their surface. Reagents for such isolation are currently available as commercial kits using columns or microbeads, e.g., ExoQuick^®^ and ExoTEST^®^. Similarly, in this method, the obtained exosomes can be subjected to further research. However, its disadvantages include the small test sample volume and that its effectiveness is dependent on the presence of specific markers [54]. The latter can significantly limit the utility of this approach, as EVs were found to be heterogeneous, with particular surface markers shared among various EVs subtypes [108]. This especially undermines the use of a single marker, e.g., CD63, which is also expressed on EVs secreted in some tumors [109]. Thus, multi-marker solutions are superior to single marker targeting. Logozzi et al. utilized an ExoTEST^®^ assay for detection and quantification of exosomes expressing CD63, Rab-5b, and caveolin-1. While a higher amount of CD63-positive and caveolin-1-positive exosomes were found in melanoma patients, detection based on caveolin-1-positive exosomes was more sensitive than that targeting CD63 [110].

In the context of melanoma, the team of Sharma et al. used an immunoaffinity approach for the capture of melanoma-derived exosomes from plasma, based on a monoclonal antibody highly specific for the CSPG4 epitope expressed by melanoma cells. Only melanoma-derived exosomes presented its expression, making them useful biomarkers of melanoma progression [111].

## 4. Clinical Relevance of Liquid Biopsy in Melanoma

### 4.1. Melanoma Detection and Prognosis

The available data suggest that enumeration of CMCs is a suitable tool for melanoma detection, allowing evaluation of its stage and prognosis. Unfortunately, the results are not consistent since various approaches are implemented by different study groups to overcome the problems of CMC rarity and heterogeneity.

Nevertheless, a few studies have already been performed proving that presence of CMCs is clinically relevant and carries prognostic value. Freeman et al., using immunomagnetic enrichment and combination of MCAM, melanoma-associated chondroitin sulfate proteoglycan (MCSP), ABCB5 and CD271 markers, showed that significantly more patients harbored CMCs in comparison to healthy individuals. However, single CMCs were also found in the control group. More importantly, there was a difference in CMC counts between metastatic and non-metastatic patients, making CMC enumeration possibly helpful for determining disease progression [70]. Studies by Ulmer et al. also showed that the number of CMCs was associated with occurrence, aggressiveness, and stage of the disease. The relation likewise applied to the overall survival (OS) of metastatic patients [112]. Similarly, differences in length of OS were observed between groups of patients harboring at least two CMCs and less than two CMCs per tested blood sample, being significantly shorter in the first case (2.0 vs. 12.1 months, respectively) [76].

Detection of tyrosinase (*TYR*) mRNA was found to be highly sensitive for identification of circulating melanoma cancer cells [80], with a study conducted by Stevens et al. reporting that the presence of *TYR* mRNA in the blood correlated with disease stage in melanoma [81]. Furthermore, a combined analysis with Breslow’s thickness and disease stage was determined as a prognostic approach for estimating disease-free survival (DFS) [113].

Analysis of multiple melanoma-associated mRNA transcripts (*MART-1*, *GalNAc-T*, *PAX-3*, and *MAGE-A3*), performed by Koyanagi et al., revealed a significant correlation between the number of positive markers and I–IV stage of the disease [114]. In turn, the outcome of a comprehensive search conducted by Khoja et al., aiming at analysis of already performed studies, was less conclusive. The results showed that CMC detection performed on I–III stage melanoma patients, based on the analysis of melanocytic transcripts (*TYR*, *MELAN-A/MART-1*, *MAGE-3*, *PMEL*) in the blood or PBMC fraction, presented great variation, with CMC outcome ranging from 13.8% [115] to 80.5% [116], thus not always being prognostically significant. Among them, the stage III patient group showed the greatest clinical utility, confirming the relation between CMC number and factors like risk of developing distant metastasis, as well as shorter DFS and OS [117]. A similar observation was made with pooled stage I–IV patients, both using melanocytic transcripts analysis and CellSearch^®^ [75,118,119]. Additionally, Hoshimoto et al. showed that the presence of more than one mRNA marker before and during the course of the treatment negatively influenced DFS and OS [118]. Interestingly, in ocular melanoma, sharing the same melanocytic markers and analyzed using the same platforms as cutaneous melanoma, five studies presented concordant results, indicating that CMCs are also prognostic factors in this type of disease [120,121,122,123,124]. PCR-based methods also proved to be helpful for identifying groups of patients with a higher risk of recurrence after lymph node removal due to the presence of metastases. The expression of mRNA markers (*TYR* and *MART-1*/*Melan-A*) before the clinical onset of the disease proved to be useful for future relapse prognosis, with 83% sensitivity and 41% specificity [125].

When applying multiparametric flow cytometry, it was also presented that CMCs detected based on the limited expression of MCAM, MCSP, ABCB5, receptor activator of NF-κB (RANK), and CD271 markers were far more abundant in melanoma, especially in late-stage patients in comparison to healthy controls. This observation did not apply to early-stage patients exclusively, where marker co-expression was extremely limited. Interestingly, a comparison of CMCs with corresponding tumor tissue revealed that ABCB5 and RANK expression is more attributed to CMCs than the tumor itself. This signifies that melanoma-initiating cells are likely preferentially released to blood circulation, causing metastatic spread. Moreover, the higher number of RANK-positive cells reflected shorter progression-free survival (PFS) [126]. These findings were confirmed by another study using the same detection method, which found significantly increased expression of metastasis-associated RANK in CMCs from melanoma stage IV vs. stage I patients, demonstrating that increased RANK expression can be a marker of metastatic melanoma [127].

Flow cytometry was also used for comparative analysis of CMCs positive for nestin (NES) and CD133, molecular markers of melanoma-initiating cells, with metastatic tissue samples. The results showed a high concordance of marker expression profile between the former and the latter. Additionally, a prevalence of NES-positive CMCs was associated with tumor burden and number of metastases, also being related to shorter OS [72].

Data obtained in the course of a meta-analysis of 53 studies focused on the abovementioned immunomagnetic cell enrichment, PCR-based methods, and a cytometric approach revealed that the prevalence of CMCs correlated with disease stage in melanoma. However, various limitations of the study design largely undermine the favorable results of this analysis [128].

Similar results were obtained by other study groups using ^HB^CTC-Chip capture [129] and a filtration method [130], also showing that CMCs can be correlated with advanced disease stage.

All this taken together indicates that CMCs are a potentially reliable factor for disease diagnosis and relapse prognosis. However, the extremely high heterogeneity of this population and multiple testing platforms of different sensitivity and specificity can lead to both false-positive and false-negative results, diminishing their clinical relevance.

Since studies of Aya-Bonilla et al. showed concordance between the dynamics of CMC scores and changes in ctDNA, it is suggested that the latter can serve as a promising biomarker for melanoma monitoring [69]. While a higher concentration of ctDNA is often detected in the plasma of patients with progressive disease, destruction of cancer cells during initiation of therapy may also lead to elevated ctDNA levels [131]. Remarkably, even patients with a low tumor load prior to therapy who progressed at first follow-up already presented higher ctDNA levels at the baseline. Furthermore, an increase in ctDNA concentration at first follow-up negatively influenced the length of OS, indicating that evaluation of ctDNA levels in treatment-naïve patients can be a prognostic factor associated with OS [40].

Apart from total ctDNA concentration, specific mutation analysis in ctDNA can also be performed. Since *BRAF* mutations are present in the majority of cutaneous melanoma patients, ctDNA analysis can be of great value in their context, representing a reliable alternative to the tissue biopsy [132]. It was found that accordance between tissue and plasma *BRAF*(*V600E*) ranged from 75% [133] to 84% [134], making its quantification a reliable biomarker in melanoma. Furthermore, an abundance of *BRAF*(*V600E*) ctDNA in treatment-naïve patients was related to tumor burden, with lower concentration reflecting longer OS and PFS compared to higher concentration (27.7 vs. 8.6 months and 9 vs. 3 months, respectively) [134].

The commonness of brain metastases development in the course of melanoma emphasizes the need for finding a sensitive tool, reflecting their status, and predicting response to therapy. A study by Lee et al. evaluated ctDNA in relation to active brain metastases treated with PD-1 inhibitors—pembrolizumab or nivolumab. Prior to the treatment, ctDNA was not detected in patients with intracranial metastases and thus was not associated with their presence. Conversely, ctDNA concentration reflected the volume of extracranial disease. Furthermore, its appearance preceded or coincided with the extracranial metastatic spread [135].

Finally, ctDNA can be applied for non-typical melanoma mutation detection (wild-type patients, constituting ca. 20% of all melanoma cases) [136] and tumor-specific gene methylation detection and be used for early diagnosis of melanoma [137]. Several study results showed that the presence of hypermethylation in the promoter region of *PTEN*, *TFPI2*, *RARB2*, *MGMT*, and *RASSF1A* genes in ctDNA enabled differentiation of melanoma patients from healthy individuals [138,139,140], with elevated levels of methylated *RASSF1A* ctDNA more attributed to metastatic cases compared to early-stage melanoma [140]. Moreover, the *RASSF1A* hypermethylation, most common in melanoma [140,141], was predictive for shortened OS [140] and poor response to biochemotherapy [141,142]. Similarly, hypermethylation in the *AIM1* gene and hypomethylation of the *LINE-1* gene detected in ctDNA were related to adverse prognosis in melanoma [143]. This piece of information suggests that although DNA methylation is not restricted to tumor cells, it is attributed predominantly to cancer-related pathways and thus may deliver vital diagnostic and predictive data [44].

Despite the abovementioned findings, ctDNA is a sensitive tool, preferentially used to monitor response to treatment instead of melanoma diagnosis, and is thoroughly described in the relevant chapter of this paper (Section 4.2).

Lastly, exosomes possess enormous biomarker potential due to their tumor-originated content, which can be applied for diagnosis and tumor burden estimation, as well as for PFS prediction. The protein content of exosomes was showed to be higher in serum of melanoma patients than in healthy individuals, irrespectively of active or not evident disease. PD-1L expression on melanoma-derived exosomes was inversely associated with disease status, and they were found to be enriched with immunosuppressive proteins, most likely mediating tumor-induced immune suppression and thus hampering immunotherapy in melanoma [144]. Their tumor origin can provide a better overview of tumor heterogeneity than histological approaches since PD-1L associated with exosomes was found in the serum of all tested patients, whereas only in 67% of tumor biopsies. Moreover, exosomal PD-L1 level, although not related to tumor Breslow thickness, melanoma type, or age of the patients, was elevated in the plasma of melanoma patients compared to healthy controls [145]. The stage of melanoma did not influence the concentration of exosomes, but patients with advanced stages had a higher content of S100B and melanoma inhibitory activity (MIA) proteins per particle, which could reflect the stage, progression, and metastases [146]. Although limited, some data already exists showing that the tumor stage is represented by specific exosomal miRNAs content. Margue et al. observed the difference between exosomal miRNAs, specifically a melanocytic marker miR-211-5p, in early- vs. late-stage patients [147]. Later on, Tengda et al. and Pfeffer et al., based on the increased level of exosomal miR-106b, miR-532-5p, miR-17, miR-19a, miR-21, miR-126, and miR-149 in melanoma patients compared to a healthy control group, managed to precisely distinguish tumor thickness, different melanoma stages, metastatic from non-metastatic patients, and patients receiving pembrolizumab from naïve-treatment patients [148,149]. Another study revealed that a lower level of miR-125b was attributed to advanced melanoma, probably as a result of a disturbance in tumor cells [150]. The protein set, consisting of tyrosinase-related protein-2 (TYRP-2), very late antigen-4 (VLA-4), heat shock protein 70 (HSP70), heat shock protein 90 (HSP90), and MET oncoprotein, was termed as an exosome-specific melanoma signature and found to be increased in stage IV patients, affecting survival and metastatic spread [62]. Similarly, differential expression of vitreal miRNAs in patients with uveal melanoma was found in comparison to healthy controls. Additionally, higher content of miR-146a was also described to be present in serum, suggesting its potential to become a non-invasive biomarker of uveal melanoma [151]. Exosomes purified from lymphatic drainage after lymphadenectomy of stage III patients contained melanoma progression-related proteins, showing that RAS/RAF/MAPK pathway-related proteins are altered depending on the volume of nodal metastases. These exosomes were also suitable for the detection of melanoma-specific mutations, like *BRAF*(*V600E*), helping to promptly identify patients at risk of relapse caused by residual disease [152].

### 4.2. Assessment of Treatment Efficacy and Acquired Resistance

The disease progression can also be evaluated via the determination of treatment efficacy. CMC enumeration before, during, and after melanoma treatment could potentially provide crucial information about its outcome, as results of some studies have proven informative for the estimation of therapy response in melanoma.

Studies involving immunomagnetic cell enrichment of CMCs through targeting MCSP, MCAM, ABCB5, and CD271 markers were conducted prior, during, and after the treatments based on surgery, as well as vemurafenib, ipilimumab, or dacarbazine. Although numbers of detected CMCs at baseline were not a prognostic factor for OS or PFS, their low counts (<2 CTCs per 1 mL of blood) were related to a more efficient response to vemurafenib treatment. Moreover, a decline in CMC prevalence after initiation of vemurafenib treatment was similarly associated with a faster response to therapy and longer OS [153].

It is worth noting that the CellSearch^®^ system used for the isolation of CMCs has a great predisposition for clinical use, although currently favorable data mostly concerns patients with metastatic disease [154]. Khoja et al., using the CellSearch^®^ system, performed sequential testing of melanoma patients treated with dacarbazine, BRAF, and MEK inhibitors, or immunotherapy with a CTLA-4 monoclonal antibody. The cut-off of 2 CMCs at baseline was found to be prognostic for OS, with 2.6 months for patients harboring ≥ 2 CMCs and 7.2 months for patients harboring < 2 CMCs. Subsequent analyses during the course of the treatment showed that maintaining the number of ≥2 CMCs was predictive for a shorter OS, compared to <2 CTCs (7 vs. 10 months) [75].

Koyanagi et al., using a multi-marker mRNA assay, showed that in relapse-free patients, the number of detected CMCs’ markers significantly decreased after neoadjuvant biochemotherapy. Reversely, their presence after completed treatment was related to a significant decrease in relapse-free and OS rates, making them a potentially prognostic factor for OS [155]. Similarly, Reid et al., measuring *MLANA*, *ABCB5*, and *MCAM* transcripts in a representative group of 230 patients, found an association between the presence of the first two and disease recurrence. Furthermore, the common *MCAM* expression was attributed to patients poorly responding to therapy [156].

Gray et al., using multiparametric flow cytometry for analysis of melanoma (MCAM, MCSP) and tumor-infiltrating (ABCB5, CD271, RANK) marker co-expression, found that only RANK-positive cells reflected the effectiveness of BRAF/MEK-targeted therapy with vemurafenib or dabrafenib/trametinib. Interestingly, 6–13 weeks after treatment initiation, an increase in CMC numbers was observed, with the presence of at least five RANK-positive CMCs being the indicator of shorter PFS. No such relation was observed in the case of the checkpoint inhibitor treatment, making it specific for targeted therapy and underlying the involvement of RANKL/RANK axis in inhibition of MAPK kinases in melanoma [126]. Corresponding research by Khattak et al. showed that the majority of RANK- and ABCB5-positive CMCs also presented PD-L1 expression. However, PD-L1-expressing CMCs were, on the contrary, associated with an effective response to treatment with PD-1 inhibitor pembrolizumab and longer PFS comparing to PD-L1-negative CMCs (26.6 vs. 5.5 months, respectively). The total number of CMCs was not an indicator of response to treatment or correlated with survival, making PD-L1-positive CMCs an independent prognostic factor. Lastly, both total CMCs and PD-L1-positive CMCs decreased after 6–12 months after treatment initiation in the majority of responders and increased or stayed at the same level in non-responders [157].

A previously quoted meta-analysis by Mocellin et al. remains in accordance with the abovementioned results, showing that CMC number was associated not only with TNM stage but also with both reduced OS and PFS. Nevertheless, the inconsistency in study designs and lack of standardization of detection methods resulted in substantial statistical variability [128].

Other technologies have also been applied in terms of treatment efficacy evaluation. An antibody-functionalized microfluidic platform for CMC capture was found to be useful for monitoring the efficacy of treatment with BRAF inhibitors in patients bearing the *BRAF*(*V600E*) mutation. The decrease was found after the treatment, and CMC counts were associated with the detailed clinical status of the disease, indicating the character of the response [129].

Finally, some innovative approaches attract attention, such as CTC-derived xenografts (CDX) from individual patients. First, in vitro experiments showed that xenografts injected in mice mirrored histopathologic and immunohistochemical characteristics of a patient’s tumor. Most importantly, CDX-bearing mice, similarly to the patient, developed molecularly compatible human melanoma metastases and similar responses, or lack thereof, to the same treatment with vemurafenib and dabrafenib. Thus, CDXs could be a future-proof tool for drug efficacy testing, predicting response to treatment and helping to drive personalized treatment decisions for advanced-stage melanoma patients [158].

In contrast to differentiated results regarding CMC use in treatment monitoring, ctDNA detection was found to strongly correlate with the clinical status of the patients, according to multiple studies. An association was presented between ctDNA and tumor burden [92,159,160], manifested via CT scans and serological markers like lactate dehydrogenase (LDH), S100B, and MIA. The advantage of ctDNA detection for melanoma tracking is its tumor origin, which is not affected by non-specific inflammatory conditions. This designates ctDNA as a reliable liquid biopsy biomarker for clinical cancer management [134].

These findings are especially valuable in terms of the assessment of *BRAF*(*V600E*) mutation for monitoring of patients treated with BRAF/MEK inhibitors. Girotti et al. showed that ctDNA reflected the response to treatment with dabrafenib/trametinib. The concentration of *BRAF*(*V600E*) ctDNA decreased along with tumor reduction and increased in a case of relapse. Higher ctDNA levels also mirrored the lack of response to other therapeutic agents, such as ipilimumab. Moreover, monitoring of ctDNA levels was predictive for treatment outcome, reflecting unsuccessful treatment with ipilimumab one week prior to CT scan and successful treatment with dabrafenib/trametinib six weeks prior to CT scan. Importantly, with the use of the WES method, the resistance mechanism was also determined. Despite BRAF inhibition, the MAPK pathway can be activated due to mutations in the *NRAS* gene, promoting tumor survival [161]. Using a panel of ten loci, ctDNA-targeted sequencing managed to detect *NRAS*(*Q61K*), *NRAS*(*Q61R*), and *PIK3CA*(*E545K*) mutations, known to mediate resistance to BRAF/MEK inhibitors, even several weeks before the scan [158].

The beneficial impact of ctDNA testing in terms of treatment monitoring was confirmed by other study groups with the use of PCR-based methods. Quantification of *BRAF*(*V600E*) ctDNA prior to and during the course of treatment with dabrafenib and vemurafenib mirrored its outcome, decreasing significantly at the moment of the strongest response while increasing significantly in the event of progression [134]. Moreover, the groups of Schreuer et al. and Grey et al. obtained concordant results [45,133], the latter additionally showing that, even in patients defined as non-responders, reduced levels of ctDNA were associated with extended PFS, indicating a positive effect of the treatment. Furthermore, baseline ctDNA concentrations were associated with subsequent response to therapy in patients treated with BRAF/MEK inhibitors (dabrafenib, trametinib, vemurafenib), as well as the length of PFS. However, some patients with higher baseline ctDNA concentrations were treatment-responders and had a PFS longer than six months, underlining the fact that a low level of ctDNA at the baseline, although prognostic, is not a unique indicator of a clinically beneficial response to treatment [45]. In patients with acquired resistance to BRAF inhibitors, mutations in the *NRAS* gene (*NRAS*(*Q61K*) and *NRAS*(*Q61R*)) have been detected and positively correlated with an increase in *BRAF* mutant ctDNA [45].

Brain metastases occur in the majority of melanoma patients, posing a significant clinical challenge. Thus, determination of a ctDNA predictive value in their management is crucial. Gray et al. revealed that the development of brain metastases was the only exception when the measurement of ctDNA was not conclusive for progression detection [45]. Similarly, in-depth studies of Lee et al. found that ctDNA cannot be treated as a reliable tool in the case of intracranial response evaluation, since neither baseline nor on-therapy ctDNA predicted the intracranial response rate [135]. This phenomenon can be theoretically explained by the fact that the blood-brain barrier can hamper ctDNA passage to the bloodstream and thus undermine its clinical value [162]. On the other hand, brain metastatic patients with undetectable ctDNA prior to and during the therapy presented improved OS compared to those with detectable ctDNA levels (39.2 vs. 10.6 months and 39.2 vs. 9.2 months, respectively), regardless of intracranial treatment response. Interestingly, the OS of patients with brain metastases and undetectable ctDNA was comparable to the OS of patients with extracranial metastases and detectable ctDNA caused by a lower intracranial response to treatment [135].

Results of Ascierto et al., obtained with the use of BEAMing technology, confirmed the abovementioned data, finding a relation between baseline and follow-up *BRAF*(*V600E*) ctDNA levels and tumor burden, response to treatment with BRAF inhibitors, prediction of disease progression prior to CT scan, and PFS. Furthermore, the detection of resistance mutations successfully predicted the development of resistance to BRAF inhibitor therapy [163].

The study of Santiago-Walker et al., collecting data from four clinical trials and a large study cohort of 836 patients, seems to conclude the abovementioned findings. *BRAF* mutations in ctDNA of late-stage melanoma patients were consistent with more than 75% of *BRAF* mutated tumors. The presence of ctDNA, carrying *BRAF* mutations, was clinically relevant and predicted a worse course of the disease. Based on this, ctDNA was defined as a reliable prognostic tool for monitoring of response to targeted treatment in melanoma patients [164].

Detection of ctDNA was proved also to be suitable for monitoring of patients receiving immunotherapy. Research by Valpione et al., conducted on metastatic melanoma patients, confirmed that baseline ctDNA concentration reflected tumor burden, with their ratio possessing a prognostic value. Increased ctDNA concentration correlated with shorter OS (8.5 vs. 22.7 months) and risk of death. A significant correlation was also found between changes in ctDNA level and tumor burden observed in the course of treatment with CTLA-4 (ipilimumab) and PD-1 (nivolumab) inhibitors [40,165]. A low level of ctDNA in treatment-naïve patients was also linked to effective response in patients treated with ipilimumab, nivolumab, and pembrolizumab and improved PFS. However, the decrease in ctDNA after the commencement of therapy was not often observed in patients receiving immunotherapy. Nevertheless, while some of those that presented substantial ctDNA decrease were qualified as non-responders, the disease was stable for the next six months, indicating that ctDNA somehow reflected the effectiveness of the treatment. Lack of response to immunotherapy, following the failure of BRAF/MEK inhibitors administration, was concordant with the persistent presence of ctDNA during and at the end of treatment [45]. Similarly, longitudinal testing of ctDNA in patients treated with CTLA-4 and PD-1 inhibitors revealed that patients presenting undetectable levels of ctDNA after initiation of treatment had a higher response rate, PFS, and OS in comparison to those with detectable levels, irrespective of baseline ctDNA status [40,166,167]. Importantly, ctDNA as a time-dependent variable was superior to LDH testing in predicting 12-month survival [166]. In addition, ctDNA profiles in patients treated with anti-PD-1 antibody allowed differentiation of patients with pseudo-progression from patients with true disease progression with a sensitivity of 90% and specificity of 100%, showing higher positive and negative values than LDH [168]. Identification of pseudo-progression, defined as infiltration of the tumor by lymphocytes, giving the impression of an enlarged tumor in CT scans, is both challenging and of great importance in this group of patients since it can prevent termination of potentially effective treatment and may guide further therapeutic decisions [44]. ctDNA was also found to be a better prognostic factor than LDH in the studies conducted by Chang et al. [169] and Valpione et al. [165], as well as better than S100 in the studies by Váraljai et al. [93] and Braune et al. [160].

All this taken together indicates that detection of tumor-associated ctDNA is a reliable and simple approach for the evaluation of response to treatment, including resistance evolution.

Treatment response can also be assessed with the use of exosomes. It was demonstrated that the level of exosomal PD-L1 differed along the course of anti-PD-1 treatment and assisted in the stratification of patients into groups of responders and non-responders for anti-PD-1 therapy. A higher level of exosomal PD-L1 in serum was attributed to non-responding patients and progression, while a lower level was linked to response to treatment. This suggested that adaptive upregulation of PD-L1 expression in exosomes could impede proliferation and cytotoxicity of T cells, evading anti-tumoral immune response [145,170]. Similarly, a decrease in exosomal PD-L1 mRNA reflected a response to anti-PD-1 treatment and was consistent with CT scan results [171]. Conversely, an increase in exosomal PD-1 was characteristic for patients responding to anti-CTLA-4 therapy with improved OS and PFS. Results obtained in respect to PD-1 and CTLA-4 indicate exosomes as effective biomarkers for monitoring of immune checkpoint inhibitor-based therapies [172]. Furthermore, in the serum of patients harboring the *BRAF*(*V600E*) mutation, exosomal miRNA content differed before and after therapy with BRAF/MEK inhibitors. Particularly, an increase of let-7g-5p and miR-497-5p miRNAs was noted during the therapy, corresponding to treatment response and prolonged PFS, respectively [173]. It was also speculated, based on in vitro studies, that melanoma-derived exosomes were also involved in resistance acquisition against BRAF inhibitors, with their participation mediated by the transport of resistance driver—platelet-derived growth factor β (PDGFRβ) to adjacent BRAF inhibitor sensitive cells [65] or by the melanocyte inducing transcription factor (MITF)-mediated upregulation of miR-211-5p, known to reduce sensitivity to BRAF inhibitors [174]. However, further research is needed to confirm this theory in the clinical setting.

### 4.3. Completed and Ongoing Clinical Trials

Liquid biopsy is not widely used in the clinical practice of melanoma due to insufficient validation of research results. The most up-to-date source of completed and ongoing clinical trials using liquid biopsy in melanoma is the ClinicalTrials.gov database [175]. Table 1 summarizes completed studies on CMCs and ctDNA in melanoma, while Table 2 presents those that are still ongoing. During recent years, some of the studies also included the detection of EVs such as exosomes as a promising source of clinically relevant information.

The high heterogeneity of CMCs and the multitude of used detection methodologies among different study groups impede the widespread application of CMCs as a biomarker of melanoma in clinical practice. Nevertheless, there are some completed and ongoing clinical trials aiming at better standardization and future implementation of CMCs detection and characterization technologies.

Several clinical studies related to CMCs describe their level before and after the use of immunotherapy, especially BRAF inhibitors. The research conducted at CHU de Nice, France, was aimed at defining a group of patients with positive CMC detection and finding the relationship between the number of CMCs and their prognostic significance. The study implemented the CellSearch^®^ system to elucidate the differences in patient survival in relation to the number of CMCs (Table 1). A similar observational, prospective pilot study based on the same assumptions is currently being conducted by Vanna Chiarion-Sileni (Padova, Italy), determining the change of CMC number before and after treatment in the whole blood of metastatic melanoma patients (Table 2).

Another study worth mentioning is the comparison of the CellSearch^®^ system and viable cells detecting S100-EPISPOT technology (Table 1). Detection of CMCs compared among patients with advanced melanoma and the control group revealed that even though the CellSearch^®^ method turned out to be more prognostic (only in this case a significant relationship with the overall survival was shown), S100-EPISPOT was significantly more sensitive, as the percentage of patients harboring ≥2 CMC was higher. The higher sensitivity of this technology indicates its likely potential for early detection of recurrence and treatment monitoring [20].

Interestingly, there is an ongoing in vivo clinical trial being conducted at the University of Arkansas. The scientists are using the photoacoustic flow cytometry (PAFC)-based prototype method to detect CMCs in three study groups consisting of healthy individuals, patients with advanced melanoma, and patients with early melanoma. The control group serves to establish the baseline. The second group is used to validate the method and the last to test the method’s ability to find CMC below the detection limit. The study is in the recruitment phase and hopefully will provide some valuable data in the near future (Table 2).

Moreover, the available studies include a comparison of CMC immunophenotyping and analysis of somatic melanoma DNA mutations. The predominant aim was the evaluation of CMC isolation with the TrueCells technology and the correlation of the results with those of immunofluorescence staining (Table 1).

ctDNA has also been a subject of clinical trials, both completed and still ongoing. A study based on samples from patients with advanced melanoma (stage IV) analyzed with the next generation sequencing technology (Sequenom Mass Array) examined the ctDNA mutations before and after chemotherapy. The additive value of this study is a double analysis conducted after the first chemotherapy administration and following three months, which provides information about treatment response (Table 1).

As far as ongoing clinical trials are concerned, there are a few notable examples. The CAcTUS pilot study (Table 2) is expected to provide information on changes in ctDNA levels, which will be used to define the moment of the switch from targeted therapy to immunotherapy. Continuing, the relationship between the presence of circulating tumor DNA and plasma levels of kinase inhibitors in patients with advanced *BRAF*(*V600*) mutant melanoma is being investigated in a study entitled “Therapeutic Drug Monitoring of BRAF-mutated Advanced Melanoma” (Table 2). Metastatic melanoma is frequently treated with a combination of anti-BRAF and anti-MEK tyrosine kinase inhibitors. The aim of the OPTIMEL study is to investigate the possibility of metastatic melanoma monitoring with the measurement of ctDNA levels in thirty-five patients with advanced melanoma treated with a combination of dabrafenib and trametinib. The next clinical trial, titled “Use of Exome Sequence Analysis and Circulating Tumour in Assessing Tumour Heterogeneity in BRAF Mutant Melanoma”, concerns the evolution of BRAF melanoma in response to vemurafenib or dabrafenib. It will determine the development of tumor resistance against these drugs based on ctDNA analysis.

## 5. Conclusions

Liquid biopsy allows crucial clinical information to be obtained using a significantly less invasive approach. Hence, the use of the described biomarkers in routine melanoma diagnosis and control of systemic therapy in melanoma patients should be considered. CMCs may be an indicator of residual disease, thus signalizing a worse prognosis for positive patients [176]. Moreover, using the information collected from CMCs, ctDNA, and mRNA/miRNA, it is possible to observe real-time changes in the tumor based on CMC number, their gene expression, and possible mutations [177]. Furthermore, quantification of S100 proteins could potentially be used in the diagnosis, monitoring, and treatment of melanoma [178]. Finally, the research into cancer exosomes has the potential to help to understand the mechanisms of drug resistance, potentially aiding the improvement of treatment effectiveness.

## Figures and Tables

**Table 1:** Completed clinical studies on circulating melanoma cells and circulating tumor DNA (based on ClinicalTrials.gov (accessed on 7 June 2021)).

No.	NCT No.	Study Title	Cancer	Location	Participants (Active/Original)
1.	NCT01573494	Study of Circulating Tumor Cells Before and After Treatment in Patients With Metastatic Melanoma	Metastatic melanoma	CHU of Nice, Nice, France	30/30
2.	NCT01558349	Circulating Tumor Cells and Melanoma: Comparing the EPISPOT and CellSearch Techniques	Metastatic melanoma	CHU of Montpellier, Montpellier, France CHU of Nîmes, Nîmes, France	73/82
3.	NCT01528774	Culture and Characterization of Circulating Tumor Cells (CTC) in Melanoma and Other Cancers	Melanoma and other cancers	Comprehensive Cancer Centers of Nevada Las Vegas, Nevada, United States	150/1000
4.	NCT02133222	Circulating Cell-free DNA in Metastatic Melanoma Patient: Mutational Analyses in Consecutive Measurement Before and After Chemotherapy	Metastatic melanoma	CHU of Nice Nice, France	22/20
5.	NCT03007823	High-Activity Natural Killer Immunotherapy for Small Metastases of Melanoma	Metastatic melanoma	Fuda Cancer Institute of Fuda Cancer Hospital Guangzhou, Guangdong, China	20/20
6.	NCT02768207	A Study to Detect V-Raf Murine Sarcoma Viral Oncogene Homolog B1 (BRAF) V600 Mutation on Cell-Free Deoxyribonucleic Acid (cfDNA) from Plasma in Participants with Advanced Melanoma	Metastatic melanoma	UZ Brussel, Brussel, Belgium Institute Jules Bordet, Brussel, Belgium CHIREC Edith Cavell, Brussel, Belgium (and 11 more…)	40/208
7.	NCT02251314	Use of Exome Sequence Analysis and Circulating Tumor in Assessing Tumor Heterogeneity in BRAF Mutant Melanoma	BRAF-mutated melanoma	Princess Margaret Cancer Centre Toronto, Ontario, Canada	12/6

**Table 2:** Ongoing clinical studies on circulating melanoma cells and circulating tumor DNA (based on ClinicalTrials.gov (accessed on 7 June 2021)).

No.	NCT No.	Study Title	Cancer	Location	Participants (Estimated)
1.	NCT01776905	In Vivo Real-Time Detection of Circulating Melanoma Cells	Melanoma stage I–IV	University of Arkansas for Medical Sciences Little Rock, Arkansas, United States	75
2.	NCT03808441	CAcTUS—Circulating Tumour DNA Guided Switch	Metastatic melanoma	The Christie NHS Foundation Trust Manchester, United Kingdom	40
3.	NCT03416933	Therapeutic Drug Monitoring of BRAF-Mutated Advanced Melanoma	Metastatic melanoma	Hôpital de Mercy, Ars-Laquenexy, Fr CHRU Nancy, Vandœuvre-lès-Nancy, Fr Institut de Cancérologie de Lorraine (ICL), Vandœuvre-lès-Nancy, Fr	35
4.	NCT03797053	Ex Vivo Expansion of Circulating Tumor Cells as a Model for Cancer Predictive Pharmacology	Melanoma stage III–IV	Saint-Louis Hospital Paris, France	450
5.	NCT01565837	Concurrent Ipilimumab and Stereotactic Ablative Radiation Therapy (SART) for Oligometastatic but Unresectable Melanoma	Melanoma stage III–IV	Comprehensive Cancer Centers of Nevada Las Vegas, Nevada, United States	50
6.	NCT02862743	Molecular Characterization of Advanced Stage Melanoma by Blood Sampling	Metastatic melanoma	CHU of Reims Reims, France	80
7.	NCT01878396	Circulating Melanoma Cells in Metastatic Patients Treated with Selective BRAF Inhibitors	Metastatic melanoma	Istituto Oncologico Veneto IRCCS Padova, Italy	200
8.	NCT02583516	Clinical Trial to Evaluate the Efficacy of Vemurafenib in Combination with Cobimetinib (Continuous and Intermittent) in BRAFV600-Mutation-Positive Patients With Unresectable Locally Advanced or Metastatic Melanoma	Melanoma stage III–IV	Hospital Universitario Donostia, San Sebastián, Guipuzcoa, Spain Hospital General Universitario Santa Lucía, Cartagena, Murcia, Spain Hospital Clínic de Barcelona, Barcelona, Spain (and 15 more…)	70
9.	NCT03175432	Bevacizumab and Atezolizumab with or without Cobimetinib in Treating Patients with Untreated Melanoma Brain Metastases	Metastatic melanoma	MD Anderson Cancer Center Houston, Texas, United States	60
10.	NCT03873818	Low-Dose Ipilimumab With Pembrolizumab in Treating Patients with Melanoma that has Spread to the Brain	Metastatic melanoma and other cancers	MD Anderson Cancer Center Houston, Texas, United States	30
11.	NCT02537600	Vemurafenib and Cobimetinib Combination in BRAF Mutated Melanoma with Brain Metastasis	Metastatic melanoma	CHU of Bordeaux, Bordeaux, France CHU Ambroise Paré, Boulogne, France CHU Brest Hôpital Morvan, Brest, France (and 14 more…)	43
12.	NCT02673970	Biomarkers for the Activity of Immune Checkpoint Inhibitor Therapy in Patients with Advanced Melanoma	Metastatic melanoma	UZ Brussel Jette, Brabant, Belgium	200
